# A robust method for calibration of eye tracking data recorded during nystagmus

**DOI:** 10.3758/s13428-019-01199-0

**Published:** 2019-03-01

**Authors:** William Rosengren, Marcus Nyström, Björn Hammar, Martin Stridh

**Affiliations:** 1 Department of Biomedical Engineering, Lund University, Lund, Sweden; 2 Humanities Laboratory, Lund University, Lund, Sweden; 3 Department of Ophtalmology, Lund University, Lund, Sweden

**Keywords:** Eye tracking, Nystagmus, Calibration

## Abstract

Eye tracking is a useful tool when studying the oscillatory eye movements associated with nystagmus. However, this oscillatory nature of nystagmus is problematic during calibration since it introduces uncertainty about where the person is actually looking. This renders comparisons between separate recordings unreliable. Still, the influence of the calibration protocol on eye movement data from people with nystagmus has not been thoroughly investigated. In this work, we propose a calibration method using Procrustes analysis in combination with an outlier correction algorithm, which is based on a model of the calibration data and on the geometry of the experimental setup. The proposed method is compared to previously used calibration polynomials in terms of accuracy, calibration plane distortion and waveform robustness. Six recordings of calibration data, validation data and optokinetic nystagmus data from people with nystagmus and seven recordings from a control group were included in the study. Fixation errors during the recording of calibration data from the healthy participants were introduced, simulating fixation errors caused by the oscillatory movements found in nystagmus data. The outlier correction algorithm improved the accuracy for all tested calibration methods. The accuracy and calibration plane distortion performance of the Procrustes analysis calibration method were similar to the top performing mapping functions for the simulated fixation errors. The performance in terms of waveform robustness was superior for the Procrustes analysis calibration compared to the other calibration methods. The overall performance of the Procrustes calibration methods was best for the datasets containing errors during the calibration.

## Introduction

Eye tracking is a useful tool to record and study eye movements. However, the nystagmus eye movements disturb the calibration procedure for individual recordings, causing comparisons of waveforms between recordings unreliable. For example, the calibration protocol assumes an ability to fixate the gaze, which is limited in people with nystagmus. Using the default calibration protocol may lead to unreliable eye tracker data, which in turn may misrepresent or even invalidate data analysis. In this paper, we explore the problems associated with calibration and propose a method that secures a repeatable and reliable gaze estimation, referred to as *point-of-regard* (PoR), which is crucial for detailed computer based nystagmus diagnostics and objective evaluation of treatment effects between recordings.

### Description of nystagmus

Nystagmus could be a symptom of an underlying oculomotor disorder, which causes involuntary movements of the eye(s) and the condition may lead to decreased visual acuity (Hertle, 2010; Hussain, 2016). There are two broad types of nystagmus: *early-onset nystagmus* and *acquired nystagmus* (Hussain, 2016; McLean, Proudlock, Thomas, Degg, & Gottlob, 2007), where the former condition is developed in the months after birth and the latter is developed later in life (Dunn, 2014). The eye movement pattern, sometimes referred to as a *waveform*, can be classified into different categories and there are at least 12 different types of nystagmus waveforms according to a classification study (Hussain, 2016; Theodorou & Clement, 2016; Dell’Osso & Daroff, 1975).

Different treatments strategies, for instance drug treatment (McLean et al., 2007) and surgery (Kumar, Shetty, Vijayalakshmi, & Hertle, 2011), have been suggested to improve the visual acuity in people with nystagmus. In order to evaluate the different strategies, eye movements before and after the treatment can be studied. Treatment effects are difficult to asses in detail without an objective evaluation of the eye movements, since people with nystagmus are often considered to be hard to diagnose by clinicians (Hussain, 2016).

Nystagmus can also be found in visually healthy subjects. *Optokinetic nystagmus* (OKN) is a reflex found in humans (Naegele & Held, 1982), which causes oscillatory eye movements similar to the oscillations found in some forms of nystagmus such as pure jerk nystagmus. It can easily be elicited by keeping the head still in a moving environment (Naegele & Held, 1982).

### Calibration of a camera based eye tracker

Nystagmus eye movements can be studied in detail with the use of an *eye tracker*. The video-based eye tracker, referred to as video-oculography (VOG) (Holmqvist et al., 2011), records eye movements using eye images captured by an infrared camera. The data from the VOG system are in this work obtained by finding the pupil center (PC) and the reflection off the cornea caused by an infrared illuminator, called the *corneal reflection* (CR). The vector between the PC and CR positions is a measure called the *pupil-corneal reflection vector* (PCRV). This measure is unique for each eye orientation and can therefore be used to estimate the PoR. In order to do this estimation from the PCRV, a relationship between the PCRV data and the corresponding PoR data is needed. The process to identify this relationship is referred to as *calibration*, which is dependent on the geometry of the experiment as well as the individual eye anatomy of each participant (Holmqvist et al., 2011).

The goal of the calibration is to find a *mapping function* (MF), e.g. a polynomial, which describes the relationship between the PCRV data and the PoR data. By presenting targets at known positions during an experiment, referred to as *calibration targets*, and simultaneously recording the corresponding PCRV data, it is possible to estimate the mapping function parameters. The number of calibration targets can vary, but common choices are 2, 5, 9, 13 and 16 targets (Holmqvist et al., 2011).

**Fig. 1:**
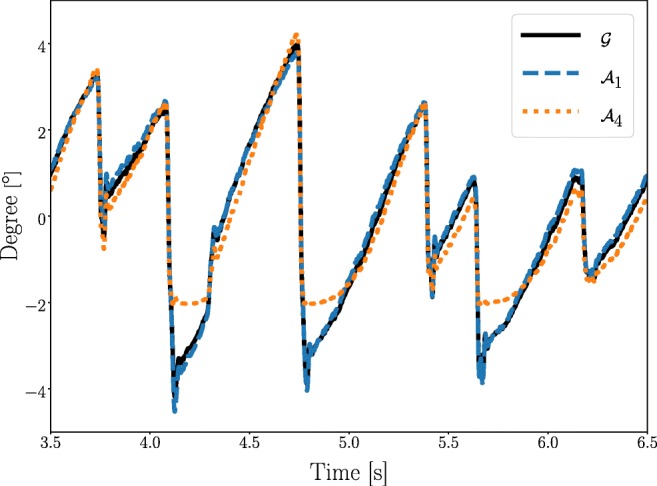
PoR estimation examples. Illustration of three different PoR polynomial estimations (see Barot, McLean, Gottlob, & Proudlock, 2013; McLean et al., 2007; Sheena & Borah, 1981 and Eqs. 5, 8 and 7), of the same recorded PCRV data. The data is obtained from a healthy participant viewing an OKN-stimulus, generating oscillatory eye movements. When comparing the three PoR estimations, it is apparent that $\mathcal {A}_{4}$ is different from the other two

The structure of the mapping function needs to be determined before its parameters can be estimated. The selection of the structure is difficult and the choice may significantly affect the resulting PoR estimation. This is illustrated in Fig. 1, where three different polynomial structures are used to estimate the same eye movement. The PoR estimations are not the same, which means that one would have to decide which of these is most likely to represent the actual eye movement.

### Previous work

Several calibration polynomials for video-based eye tracking have previously been studied. One study investigated more than 400,000 polynomials and evaluated their performance based on the *average error* (accuracy), *maximum error*, *standard deviation* of the estimated PoR, *number of polynomial parameters* and *head movement tolerance* (Cerrolaza, Villanueva, & Cabeza, 2008). Another study tested polynomial structures based on accuracy and the number of calibration targets (Blignaut & Wium, 2013). The two studies were using simulated data or data from participants with no visual impairments. In both Cerrolaza et al., (2008) and Blignaut and Wium (2013), *accuracy* was used to evaluate the calibration MFs. As is pointed out perfect accuracy, or goodness of fit, can be achieved by using the same model order as the number of calibration targets (Blignaut & Wium, 2013). The calibration polynomial is, however, used also for other gaze positions and should be tested also for these (Blignaut & Wium, 2013).

#### Previous work on nystagmus calibration

Different approaches for *calibration data selection* for nystagmus applications have previously been published. This is an important part of the calibration since the selected calibration data should represent that the participant looked at the displayed calibration target. If the selected calibration data do not represent the “correct” fixation, there is a risk of misrepresenting eye movement data.

A method to find saccades in eye movement data based on adaptive acceleration thresholds was presented in Behrens, Mackeben, and Schröder-preikschat (2010). The intent of the method was not calibration of nystagmus data, but it served as the basis for the development of a method designed for the nystagmus case. The nystagmus specific version identified the slowest eye movement velocities, referred to as *foveation periods* (Dunn, 2014). The method is based on an algorithm for saccade detection in uncalibrated data, which is used to divide the waveform into fast and slow eye movements. The foveations are found in the slow phase of the data. Another approach to find foveations was presented in (Dell’Osso, 2005), where manual annotation to mark the start and end times of the foveations, was used. While there has been some work on how calibration data are selected, literature on the suitability of various polynomials for nystagmus recording purposes is sparse.

Many papers concerning nystagmus and eye tracking do not report how calibration was performed and evaluated. In Table 1, nine different studies are summarised. As can be seen from the table, only three of the nine studies (McLean et al., 2007; Dunn, 2014; Barot et al., 2013) report any sort of calibration model structure, although the calibration MF details are not explicitly presented in any of the papers. Only three of the studies (Dunn, 2014; Abel, Wang, & Dell’Osso, 2008; Dell’Osso et al., 2011) report any type of data quality measure or accuracy. In the two first, the reported accuracies are taken from the manufacturer’s specification sheet and therefore reveals no information about the accuracy for participants in these particular studies.

**Table 1:** Summary of nine different studies, their calibration and validation protocols, the calibration methods and the calibration method performance

Study	System	Calibration positions	Data selection method	Calibration polynomial	Validation	Reported data quality
McLean et al., (2007) (101)	SMI Eye Link, 250 Hz	1: 3X3 grid, 0° and ± 20° Horizontal, ± 15° Vertical; 2: 3° steps from − 24° to 24°. Start point (− 24°, − 24°), Stop point (24°, 24°)	1:Information Missing (U); 2:Fixation (U)	1: Information Missing; 2: Fourth Order Poynomial	Information missing	Information missing
Tai et al., (2010) (6)	EyeLink 1000, 500 Hz	0° and ± 10° Horizontal and Vertical	Not explicitly specified (U)	Information Missing	Information Missing	Information Missing
Abel et al., (2008) (11)	EyeLink II	Information Missing	Foveation Periods (U)	Information Missing	Information Missing	0.5°–1.0° (Manufacturer Numbers)
Barot et al., (2013) (16)	EyeLink II	30° Left to 30° Right in steps of 3°	Foveation Periods (A)	Best line of fit	Information Missing	Information Missing
Dell’Osso et al., (2011) (24)	EyeLink II, 500 Hz	Information Missing	Foveation Periods (U)	Information Missing	Information Missing	0.5°–1.0° (Manufacturer Numbers)
(Hertle et al., 2011) (19)	Ober 2 or EyeLink, 500 Hz or 1000 Hz	1° targets or 3° pictures	End of fast phase (U)	Information Missing	Information Missing	Information Missing
Taibbi et al., (2008) (28)	EyeLink II, 500 Hz	Information Missing	Foveation Periods (U)	Information Missing	Information Missing	Information Missing
Thomas et al., (2008) (56)	EyeLink 250 Hz	0° and ± 15° Horizontal and Vertical	Foveation Periods (U)	Information Missing	Information Missing	Information Missing
Dunn (2014) (1)	EyeLink 1000, (include sampling frequency)	± 5° Horizontally, ± 3° and (0°, 0°)	Automatic Foveation Algorithm (Dunn, 2014) (A)	Regression with cross term. Degree unspecified.	Self Validation	mean and standard deviation for horizontal and vertical values

All studies used an EyeLink system (except (McLean et al., 2007) which used the SMI EyeLink) and were concerned with technical, analytical or clinical applications of eye tracking and nystagmus. The columns represent from left to right: the reference to the study (with number of citations as of December 7, 2017), the eye tracker used in the study, the calibration target positions, the calibration data selection strategy, the calibration polynomial structure (note that no explicit equations are written), the validation protocol and the reported data quality. The calibration data strategy includes manually selection (M), automatic selection (A) or not explicitly stated (U)

#### Calibration polynomials

There are various references to calibration polynomials used in nystagmus eye tracking research. Four of these polynomials are evaluated in this paper. As described above, calibration data are used to estimate the polynomial coefficients where the input to the calibration polynomial is PCRV, denoted PC in the equations, data and the output is PoR data. Table 2 summarises the characteristics of the four selected polynomials previously used in the nystagmus eye tracking literature.

**Table 2:** Summary of the calibration polynomials found in eye tracking and nystagmus related studies

Study	Polynomial [***P***]	Eye tracking data vector [***u***_*PC*_]	Property
**Barot et al., (2013)	$ \mathcal {A}_{1}$ (5)	[1 *x*_*PC*_*y*_*PC*_]^*T*^	Linear mapping (Linear)
**Dunn (2014)	$\mathcal {B}$ (6)	[1 *x*_*PC*_*y*_*PC*_*x*_*PC*_*y*_*PC*_]^*T*^	Linear mapping + Rotation (non-linear)
* Stampe (1993)	$\mathcal {G}$ (7)	$[1 \quad x_{PC} \quad y_{PC} \quad x_{PC}^{2} \quad y_{PC}^{2} \quad x_{PC}y_{PC}]^{T}$	Quadratic mapping + Rotation (non-linear)
**McLean et al., (2007)	$\mathcal {A}_{4}$ (8)	$[1 \quad x_{PC} \quad x_{PC}^{2} \quad x_{PC}^{3} \quad x_{PC}^{4} \quad y_{PC} \quad y_{PC}^{2} \quad y_{PC}^{3} \quad y_{PC}^{4}]^{T}$	Fourth order (non-linear)

*:The polynomial suggested in Stampe (1993) has been slightly changed compared to the original proposal. The corner correction terms *m*[*q*] and *n*[*q*] used in Stampe (1993) are not estimated for each quadrant but rather for the entire plane. There are not any direct references in nystagmus research to this method in the literature presented in this work. Since it is a common calibration polynomial it was included.

**: Polynomials which were not explicitly stated. Instead they have been interpreted from the context

The PoR estimation, ***p***_*PoR*_ = [*x*_*PoR*_*y*_*PoR*_]^*T*^, is computed using a polynomial, ***P***, and eye tracker data, ***u***_*PC*_, as,

1 $$ \boldsymbol{p}_{PoR} = \boldsymbol{P}\boldsymbol{u}_{PC}. $$

where ***u***_*PC*_ = [*x*_*PC*_*y*_*PC*_]^*T*^. The selected structure of ***P*** determines the structure of ***u***_***PC***_ (see Table 2). The purpose of the calibration is to estimate the coefficients of the polynomial

2 $$ \boldsymbol{P} = \left[\begin{array}{l} \boldsymbol{p}_{\boldsymbol{h}} \\ \boldsymbol{p}_{\boldsymbol{v}} \end{array}\right], $$

where ***p***_***h***_ and ***p***_***v***_ are the horizontal and vertical polynomials respectively. The coefficients are estimated using a least squares solution according to

3 $$ \boldsymbol{p}_{\boldsymbol{d}} = (\boldsymbol{U}_{PC}\boldsymbol{U}_{PC}^{T})^{-1}\boldsymbol{U}_{PC}^{T} \boldsymbol{t}_{d}, $$

where *d* is either the horizontal or the vertical direction, ***U***_*PC*_ is a matrix containing the calibration data vectors for each calibration target,

4 $$ \boldsymbol{U}_{PC} = \left[\begin{array}{l} \boldsymbol{u}_{PC}(1) \\ {\vdots} \\ \boldsymbol{u}_{PC}(n) \end{array}\right], $$

***t***_*d*_ is a vector with calibration targets of direction *d*, and *n* is the number of calibration targets. The different polynomials evaluated in this work are given in the equations below:

5 $$ \mathcal{A}_{1} = \left[\begin{array}{lll} a_{0, c} & a_{0, x} & 0 \\ a_{1, c} & 0 & a_{1, y} \end{array}\right], $$

6 $$ \mathcal{B} = \left[\begin{array}{llll} b_{0, c} & b_{0, x} & b_{0, y} &b_{0, xy} \\ b_{1, c} & b_{1, y} & b_{1, y} & b_{1, xy} \end{array}\right]. $$

7 $$ \mathcal{G} = \left[\begin{array}{llllll} g_{0, c} & g_{0, x} & g_{0, y} & g_{0, x^{2}} & g_{0, y^{2}} & g_{0, xy}\\ g_{1, c} & g_{1, x} & g_{1, y} & g_{1, x^{2}} & g_{1, y^{2}} & g_{1, xy} \end{array}\right], $$

8 $$ \mathcal{A}_{4} = \left[\begin{array}{ll} a_{0, c} & a_{1, c} \\ a_{0, x} & 0\\ a_{0, x^{2}} & 0 \\ a_{0, x^{3}} & 0 \\ a_{0, x^{4}} & 0 \\ 0 & a_{1, y} \\ 0 & a_{1, y^{2}}\\ 0 & a_{1, y^{3}} \\ 0 & a_{1, y^{4}} \end{array}\right]^{T}. $$

### Aim of this paper

The aims of this paper are to propose and evaluate a new calibration MF generating consistent PoR estimations across recording sessions and participants and compare it to other calibration mapping functions previously used in nystagmus research. The main objective is to find an MF which reliably can be used to evaluate the effects of different nystagmus treatments, even when the participant fails to accurately fixate the calibration target.

## Proposed method

In this section a new calibration method is proposed. It is developed for video-based eye trackers using a nine-point calibration and a geometrical setup similar to that of an EyeLink 1000 Plus in desktop mode. The method consists of two parts: First, an outlier correction algorithm aimed at correcting inaccuracies in the recorded calibration data. Second, a linear mapping function based on *Procrustes analysis* is proposed. The method is based on 5 s of data recorded for each calibration target, as will be presented in more detail in “Calibration method evaluation”.

### The outlier correction algorithm

For the recommended setup of the eye-tracker used in this work, the horizontal data typically have the following structure; the horizontal PoR data are dependent only on the horizontal PCRV data, and not on the vertical PCRV data. Thus, horizontal PCRV for a horizontal gaze position is approximately the same, regardless of the vertical gaze position. This characteristic is used to create an algorithm to reduce errors in the calibration dataset. The algorithm is based on nine calibration targets distributed in a 3 × 3 grid where the calibration data for each calibration target are mapped to one coordinate pair. In this case there are 9 two-dimensional coordinates; one for each two-dimensional calibration target. The outlier correction algorithm consists of two stages.

#### Stage I

**Fig. 2:**
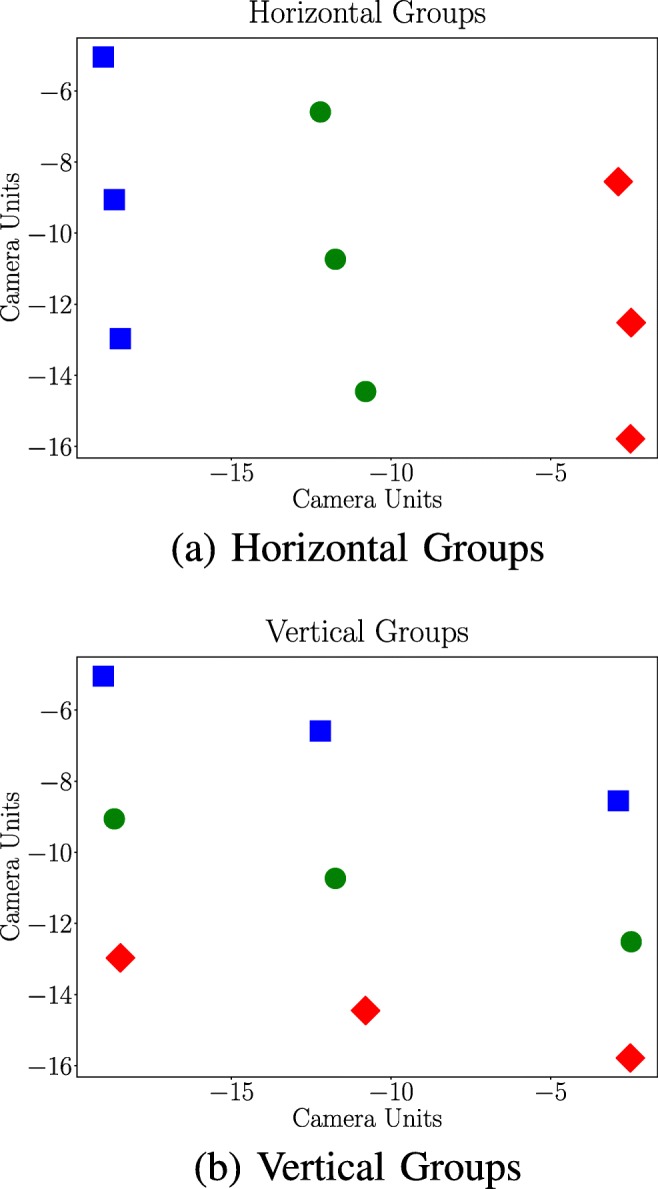
Group Division. The data points divided after the horizontal target values Fig. 2a and vertical target values Fig. 2b. All data points of the same colour and shape belong to the same horizontal group Fig. 2a or same vertical group Fig. 2b

1. Divide the data into six groups with three adjacent data points in each. Half of the groups share a horizontal calibration target value (see Fig. 2a) and the other half share the vertical calibration target value (see Fig. 2b).
2. Fit a line to the three data points in each of the six groups.
3. Compute the angle between each of the vertically fitted lines and each of the horizontally fitted lines (3 × 3 computations).
4. If the angle deviates more than 25° from the expected 90°, the vertical line is considered to contain an outlier. The value of 25° was chosen empirically.

If one or more outliers were found during Stage I, Stage II is initiated.

#### Stage II

1. An outlier is detected by finding the datapoint with the largest horizontal deviation from the vertical line.
2. Corrected coordinates of the outlier are computed as the average of the other data points on each of the intersecting horizontal and vertical lines, i.e., the new horizontal data point value is computed as the average of the corresponding horizontal data points of the vertical line, and the new vertical data point value is computed as the average of the corresponding the vertical data points of the horizontal line.

**Fig. 3:**
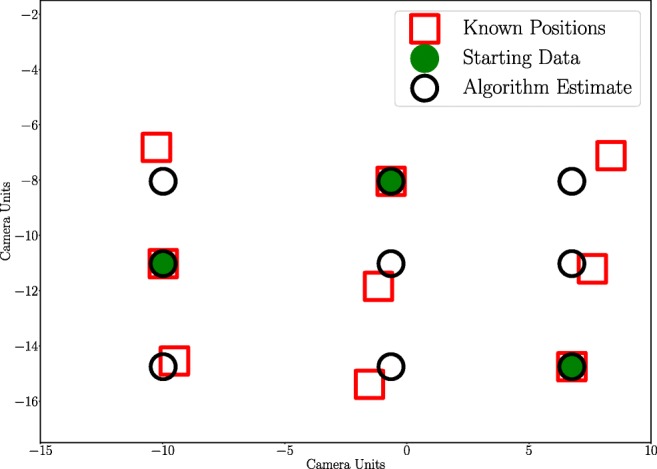
Illustration of the outlier correction algorithm where 6 out of 9 calibration data positions have been removed from the data set. The red squares represent the known calibration data positions, the green filled circles represent the known data before the algorithm estimation, and the black circles represent the estimated calibration data positions

An example of calibration data points before and after outlier correction is shown in Fig. 3.

### Procrustes calibration

In the calibration process, a set of *n* (here *n* = 9) two-dimensional data points (calibration data) are fitted to another set of *n* two-dimensional data points (calibration targets). Both of these data sets can be viewed as two-dimensional shapes, and the objective of the calibration is to identify the best transformation from the calibration data shape to the calibration target shape. In this work, *Procrustes analysis* (Gower, 1975) is used to compare and align the two datasets. Three steps are involved in the Procrustes analysis: translation, scaling and rotation. Once they have been estimated, they can be used to compute the gaze positions from PCRV data.

The three transformations have been implemented in the following way:

- Construct the calibration data matrix $\boldsymbol {D} =\left [\begin {array}{ll}\boldsymbol {x}_{d} & \boldsymbol {y}_{d} \end {array}\right ]^{T} $ as a 2 × *n* matrix where *n* is the number of calibration targets, and the calibration target matrix $\boldsymbol {T} = \left [\begin {array}{ll}\boldsymbol {x}_{t} & \boldsymbol {y}_{t} \end {array}\right ]^{T}$ contains the corresponding calibration targets.
- Center both the calibration data and calibration target datasets by subtracting their respective horizontal and vertical averages from each data set to create ***D***_***μ***_ and ***T***_***μ***_.
  9 $$ \boldsymbol{D}_{\mu} = \left[\begin{array}{ll}\boldsymbol{x}_{d} - \bar{x}_{d}\\ \boldsymbol{y}_{d} - \bar{y}_{d} \end{array}\right] = \left[\begin{array}{ll}\boldsymbol{x}_{d, c}\\ \boldsymbol{y}_{d, c} \end{array}\right], $$
  10 $$ \boldsymbol{T}_{\mu} = \left[\begin{array}{ll}\boldsymbol{x}_{t} - \bar{x}_{t}\\ \boldsymbol{y}_{t} - \bar{y}_{t} \end{array}\right] = \left[\begin{array}{ll}\boldsymbol{x}_{t, c}\\ \boldsymbol{y}_{t, c} \end{array}\right], $$
  where $\bar {x}_{d}$ is the average of ***x***_***d***_, $\bar {y}_{d}$ is the average of ***y***_***d***_, $\bar {x}_{t}$ is the average of ***x***_***t***_ and $\bar {y}_{t}$ is the average of ***y***_***t***_.
- Compute the norms, *N*_*D*_ and *N*_*T*_, using
  11 $$ N_{D} = \sqrt{\sum\limits_{i = 1}^{n}x_{d, c}^{2}(i) + \sum\limits_{i = 1}^{n}y_{d, c}^{2}(i)} $$
  where *x*_*d*, *c*_(*i*) ∈***x***_*d*, *c*_ and *y*_*d*, *c*_(*i*) ∈***y***_*d*, *c*_,
  12 $$ N_{T} = \sqrt{\sum\limits_{i = 1}^{n}x_{t, c}^{2}(i) + \sum\limits_{i = 1}^{n}y_{t, c}^{2}(i)} $$
  and *x*_*t*, *c*_(*i*) ∈***x***_*t*, *c*_ and *y*_*t*, *c*_(*i*) ∈***y***_*t*, *c*_. The datasets are scaled according to:
  13 $$ \boldsymbol{D}_{N} = \frac{\boldsymbol{D}_{\mu}}{N_{D}} $$
  14 $$ \boldsymbol{T}_{N} = \frac{\boldsymbol{T}_{\mu}}{N_{T}} $$
- The rotation, ***R***, is computed using singular value decomposition (SVD). In general, the SVD decomposes a matrix ***M*** into two orthonormal matrices ***U*** and ***V*** and a diagonal matrix ***S*** that contains the singular values *σ*_*l*_, *l* ∈ [1, *k*]. In Procrustes analysis, $\boldsymbol {M} =\boldsymbol {D}^{T}_{N}\boldsymbol {T}_{N}$.
  15 $$ \boldsymbol{D}^{T}_{N}\boldsymbol{T}_{N} = \boldsymbol{U}\boldsymbol{S}\boldsymbol{V}^{H}, $$
  where
  16 $$ \boldsymbol{R} = \boldsymbol{U}^{H}\boldsymbol{V}. $$
  and
  17 $$ \boldsymbol{S} = diag(\sigma_{1}, \ldots, \sigma_{k}). $$
- Once the translation, scaling and rotation parameters have been estimated, the PoR estimation, ***p***_*PoR*_, is computed as follows:
  18 $$ \boldsymbol{p}_{PoR} = \kappa \boldsymbol{R} \boldsymbol{p}_{PC} - \boldsymbol{L} $$
  where
  19 $$ \kappa = \frac{N_{T}} {N_{D}}\sum\limits_{i = 1}^{k}\sigma_{i}, $$
  20 $$ \boldsymbol{L} = \kappa\left[\begin{array}{l} \bar{x}_{d} \\ \bar{y}_{d} \end{array}\right]\boldsymbol{R} - \left[\begin{array}{l} \bar{x}_{t} \\ \bar{y}_{t} \end{array}\right], $$
  and
  21 $$ \boldsymbol{p}_{PC} = \left[\begin{array}{l} x_{PC} \\ y_{PC} \end{array}\right]. $$

This method is denoted as $\mathcal {P}$.

## Calibration method evaluation

In this Section the evaluation strategy of the proposed method is presented. The Section consists of three main parts; the recording of *nystagmus data* (“The nystagmus data experiment (NDE)”), the recording of *control data* (“The control data experiment (CDE)”) and the performance evaluation measures (“Comparing calibration methods”).

### Hardware and software

Binocular, raw pupil and CR data were recorded with an EyeLink 1000 Plus (desktop mode) with a sampling frequency of 1000 Hz using the host software v. 5.09 and the DevKit 1.11.571. The center of mass tracking mode was used. The eye tracker camera was placed in accordance with the recommendations of the manufacturer (SR-Research, 2010). PsychoPy (version 1.83) (Peirce, 2007) was used to present all stimuli. The stimulus was presented on an ASUS VG248QE monitor with a resolution of 1920 × 1080 pixels, with dimensions 53 *cm* × 30 *cm*. The participant to monitor distance was 80 cm.

A chin and forehead rest was used for all participants. The analysis software was written in Python (version 2.7).

### The nystagmus data experiment (NDE)

#### Participants

The nystagmus data experiment was performed with patients diagnosed with nystagmus. The diagnosis was performed by Björn Hammar (MD), senior consultant at the neuro-ophthalmology unit at Skåne University Hospital in Lund, Sweden. This dataset is denoted **NDE data**. A total of eight patients with nystagmus were recorded, two of which were recorded twice totalling ten separate recordings. Two of the participants were female and six were male. Out of the ten recordings, four were excluded from the data set; one due to lack of validation data, two due to loss of calibration data (too many blinks during the recording of calibration data) and one due to too small oscillations. For this participant, only the data from one out of the nine calibration targets consisted of oscillations with an amplitude larger than 1° and a frequency higher than 2 Hz. Out of the six remaining recordings, from five different participants, all were diagnosed with infantile nystagmus (*M* = 35.3 [year], *SD* = 15.9[year]).

#### Data recording

The experiment included calibration and validation data recordings. Both calibration and validation data were recorded monocularly for both eyes by covering one eye and recording the other eye. Nine calibration targets were presented to each patient in a randomised order. The calibration targets were placed in a 3 × 3 grid. The horizontal target positions were 0° and ± 18° and the vertical target positions were 0° and ± 10°. The validation targets were placed in a 2 × 2 grid where the horizontal and vertical validation target positions were (± 5°,± 5°) respectively. The calibration target was a black circle with radius of 0.6° with a red circle of radius 0.15° in the center. The targets were presented on a grey background. The presentation duration of each calibration target and validation target was decided manually. The goal duration for each target was 5 s (M = 5.02 [s], SD = 1.24 [s]). The experiment also included fixation, smooth pursuit, saccade and optokinetic nystagmus tasks which were not included in this work.

#### Calibration data selection

The calibration data selection algorithm presented in Dunn (2014) was implemented. Some adjustments were made to the original algorithm:

- Instead of computing saccade velocity thresholds for the entire calibration data set, the thresholds were computed for each calibration target.
- The saccade acceleration threshold was not implemented, due to too heavy saccade rejection.
- The adaptive filter to find foveations was not implemented. Instead, each slow phase longer than 50 ms was considered as a potential foveation. The first 50 ms directly after the onset of the slow phase were considered to be the most likely foveation candidate.

**Fig. 4:**
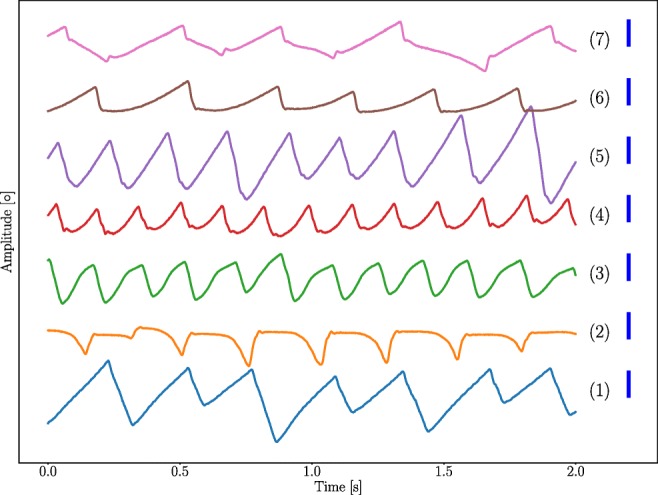
Various waveform recordings from the **NDE data** set for different participants. These are representative of the waveforms found in the dataset from the six participants. The length of blue scale bar at the right side of each signal is 4°. The calibration was preformed using the Procrustes calibration method

The observed waveforms in the NDE database are illustrated in Fig. 4.

### The control data experiment (CDE)

#### Participants

A total of eight participants were included in the dataset, one female and seven male (*M* = 37.0 [year], *SD* = 7.7 [year]). This data set is denoted **CDE data** and was divided into two subsets, see “Two **CDE** subsets”. Data from one participant was excluded due to data loss (too many blinks during the recording of calibration data).

#### Data recording

**Fig. 5:**
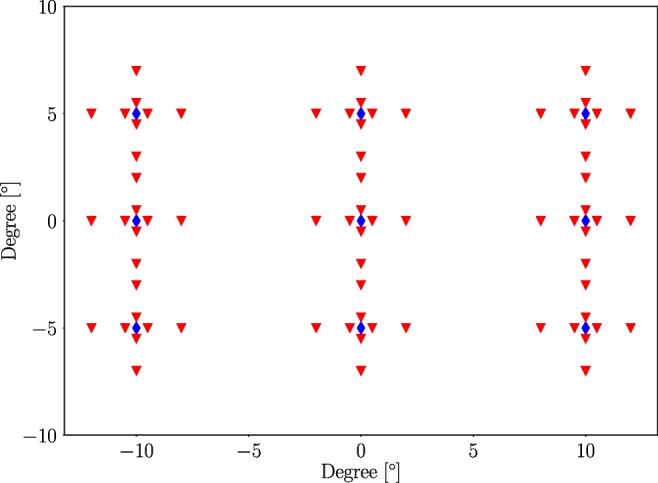
The 81 calibration targets used for the **CDE** calibration are shown. The blue diamonds represent calibration targets and the red triangles represents targets simulating fixation inaccuracies. The targets were presented in random order. Each target was shown for 1.5 seconds

The calibration protocol consisted of 81 calibration targets using a standard 3 × 3 grid with nine possible positions for each calibration target, one reference position and eight offset positions. The participants were recorded binocularly. The distribution of the targets is shown in Fig. 5. The vertical and horizontal offset amplitudes were ± 0.5° and ± 2.0°. Each target was shown for 1.5 s. The positions of the calibration targets were evenly distributed between − 10° and 10° in the horizontal direction and − 5° and 5° in the vertical direction, not counting the offsets. The calibration target was a white circle with radius of 0.6° with a black circle of radius 0.15° in the center. The targets were presented on a grey background. The calibration targets were presented in a randomised order and the offset magnitude at each calibration target was also randomised. Since no nystagmus is present in the **CDE data** a different method for calibration data selection was needed, see “Calibration data selection”.

**Fig. 6:**
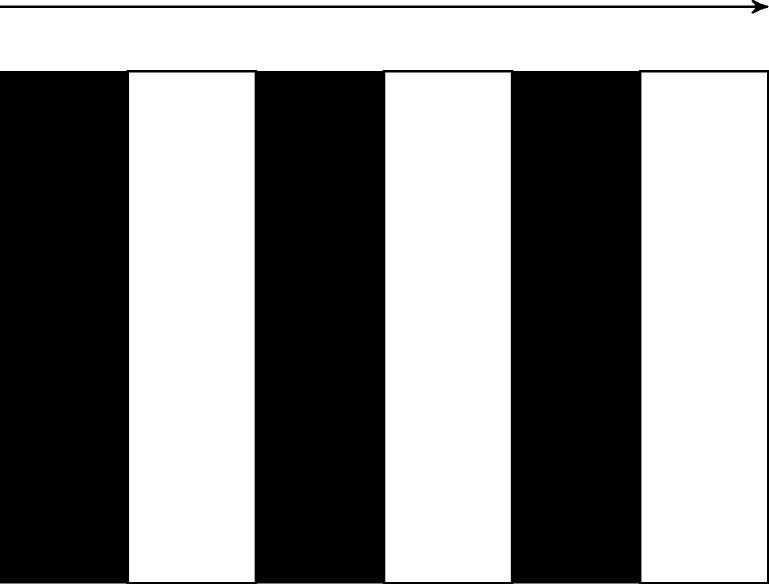
Illustration of the black and white striped pattern used to elicit nystagmus eye movement in healthy participants. The participant was asked to fixate in the center of the stripped screen when the pattern was in motion. The motion of the stimulus in the configuration illustrated above was horizontal. If the pattern is rotated 90° the stimuli moves vertically

Two OKN tasks were recorded for this dataset. A black and white striped pattern, see Fig. 6, was used to elicit OKN. In the first OKN task, the pattern was moving horizontally. In the second task, the pattern was rotated 90° and moved vertically. In both cases, the temporal frequency of the pattern was 8 cycles / second (in the moving direction), the spatial frequency was 0.5 cycles / degree and the duration was 15 s. Before the pattern was set in motion the participants were asked to look in the center of the screen and keep looking there as long as the pattern was moving. The experiment also included fixation, smooth pursuit and saccade tasks, which were not included in this work. The OKN datasets were used to compute the *calibration plane distortion* and the *waveform robustness*, described in “Calibration plane distortion & waveform robustness”.

#### Two **CDE** subsets

The **CDE data** datasets were divided into two subsets: one which contains only calibration targets with no offset, **CDE - NO**, and one which contains calibration targets with a random offset for each calibration target, **CDE - O**. The notations NO and O represent datasets with no introduced offsets and with introduced offsets, respectively. While the **CDE - NO** data correspond to data from participants without any visual impairment, the **CDE - O** data simulate potential fixation inaccuracies caused by the nystagmus oscillations for different angles during the calibration.

The **CDE - O** dataset was created by repeating the calibration data selection process 50 times, each time assigning a horizontal random error (including 0°) to each calibration target. Each repetition was independent of previous repetitions.

#### Calibration data selection

The rationale for calibration data selection at each calibration target, is that the PCRV segment with the least variance best represents a fixation. The calibration data selection method is described below:

- First, in order to avoid influence of the time it takes to change positions after a new calibration target has appeared, the first 500 ms of the recorded data for each calibration target are removed.
- Second, the 200 ms window with the smallest variance of the following PCRV data are computed. The total variance, $s_{tot}^{2}$, is computed according to Eq. 22, where ${s_{x}^{2}}$ and ${s_{y}^{2}}$ are the horizontal and vertical variance respectively.
  22 $$ s_{tot}^{2} = {s_{x}^{2}} + {s_{y}^{2}} $$
- Finally, the horizontal and vertical calibration data position estimates are computed as the averages of the 200 ms window found in step 2.

### Comparing calibration methods

In this work, three different measures are used to compare the characteristics of the different mapping functions. These are accuracy, *α*, *calibration plane distortion*, *μ*_*d*_, and *waveform robustness*, *ξ*. Accuracy is tested on a limited number of validation targets, which in this work is equal to four targets per participant. The calibration plane distortion is the distance between two PoR estimations from the same MF. Finally, the waveform robustness is computed as the difference between two PoR estimations after adjusting for the linear properties translation, rotation and scaling between the two PoR estimations.

#### Accuracy

The accuracy for validation target point *k*, *α*_*k*_, is computed according to Eq. 23 where *x*_*PoR*_(*k*) and *y*_*PoR*_(*k*) are the mapping function estimates of the horizontal and vertical validation target positions, respectively, and *x*_*s*_(*k*) and *y*_*s*_(*k*) are their corresponding known validation target positions. The accuracy computation in Eq. 23 results in one single value for each validation target. A small accuracy value means good performance, while a large value means poor performance.

23 $$ \alpha_{k} = \sqrt{({x}_{PoR}(k) - x_{s}(k))^{2} + ({y}_{PoR}(k) - y_{s}(k))^{2}} $$

The accuracy is presented in the following way. For each mapping function, the average accuracy of each eye of all validation data for one dataset is computed. This means for example that the **CDE O** dataset contain: 7 participants × 4 validation targets × 50 iterations = 1400 accuracy samples.

The accuracy is calculated separately for all three datasets. In order to evaluate the performance of the outlier correction algorithm (see “The outlier correction algorithm”), the accuracy results for the **NDE** dataset without the outlier correction algorithm are also calculated.

#### Calibration plane distortion & waveform robustness

The calibration plane distortion computations were implemented in the following way. If $\boldsymbol {P}_{PoR1} = \left [\begin {array}{ll} \boldsymbol {v}_{x} & \boldsymbol {v}_{y} \end {array}\right ]^{T}$ and $\boldsymbol {P}_{PoR2} =\left [\begin {array}{ll} \boldsymbol {w}_{x} & \boldsymbol {w}_{y} \end {array}\right ]^{T}$ are two matrices of dimension *L* × 2 containing gaze estimations, the calibration plane distortion, *μ*, is defined as:

24 $$ \mu(\boldsymbol{P}_{PoR1}, \boldsymbol{P}_{PoR2}) = \frac{1}{L}\sum\limits_{l = 1}^{L}\sqrt{{\delta^{2}_{x}}(i) + {\delta^{2}_{y}}(i)}. $$

where

25 $$ \delta_{x}(i) = v_{x}(i) - w_{x}(i); v_{x} (i) \in \boldsymbol{v}_{x}, w_{x}(i) \in \boldsymbol{w}_{x}, $$

and

26 $$ \delta_{y}(i) = v_{y}(i) - w_{y}(i); v_{y}(i) \in \boldsymbol{v}_{y}, w_{y}(i) \in \boldsymbol{w}_{y}. $$

The calibration plane distortion is used to compute how close two PoR estimations are in absolute terms, i.e., the distance on the stimuli screen. Even though it includes changes in the waveform, it is likely that translation effects are a dominant part of the calibration plane distortion value. In order to study the effects on the waveform itself, the waveform robustness measure was computed using the Procrustes Distance, *D*_*P*_. It is defined as:

27 $$ D_{P}(\boldsymbol{P}_{PoR1}, \boldsymbol{P}_{PoR2}) = 1 - \left( \sum\limits_{l = 1}^{k}\sigma_{l}\right)^{2} $$

where ***S*** = *diag*(*σ*_1_,…, *σ*_*k*_), is computed according to Eq. 15 and *D*_*P*_ ∈ [0,1]. The ***P***_*PoR*1_ and ***P***_*PoR*2_ matrices correspond to the ***D*** and ***T*** matrices described in “Procrustes calibration”.

If ***P***_*f*{*k*}, *NO*_ is a gaze estimation from mapping function *f*{*k*} from the **CDE - NO** dataset and ***P***_*f*{*k*}, *O*_ is a gaze estimation from mapping function *f*{*k*} from the **CDE - O** dataset, where $f = \{\mathcal {A}_{1}, \mathcal {B}, \mathcal {G}, \mathcal {A}_{4}, \mathcal {P} \}$ and *k* ∈ [0,4], the calibration plane distortion, *μ*_*k*_, and the waveform robustness, *ξ*_*k*_, for mapping function *k* are defined in Eqs. 28 and 29 respectively.

28 $$ \mu_{k} = \mu(\boldsymbol{P}_{f\{k\}, NO}, \boldsymbol{P}_{f\{k\}, O}) $$

29 $$ \xi_{k} = D_{P}(\boldsymbol{P}_{f\{k\}, NO}, \boldsymbol{P}_{f\{k\}, O}). $$

In order to reduce the influence of blinks and other artefacts in the OKN datasets, the blink removal algorithm used for the calibration data was applied to the OKN data before the computation of both the calibration plane distortion and the waveform robustness. The method is described in full detail in Dunn (2014).

The results for calibration plane distortion and waveform robustness are presented as empirical *cumulative distribution functions* (*CDF* s), as well as the area under each CDF curve, *A*_*CDF*_. The area computations for the calibration plane distortion were bounded to 1° as this is considered a good calibration accuracy (Hansen and Ji, 2010). The area computation for the waveform robustness was bounded to 0.2 as the results from “Waveform robustness and accuracy examples” showed that Prob(*D*_*P*_ > 0.2) ≈ 0.01 for the $\mathcal {G}$ MF. The *A*_*CDF*_ was adjusted such that *A*_*CDF*_ ∈ [0,1] by dividing the computed area with the maximum CDF-value for the area computation. Using this definition of the waveform robustness, the *A*_*CDF*_ for the Procrustes calibration method will be 1.0 be definition.

## Results

### Accuracy

The accuracies in all datasets are presented in Table 3. When comparing the accuracies for the **NDE** dataset with and without OA, it can be seen that the OA improves the accuracy at least for one of the eyes for all **mapping functions**. The most prominent improvements are seen for the **mapping functions** with a higher degree of freedom, i.e., $\mathcal {G}$ and $\mathcal {A}_{4}$. As expected, the $\mathcal {G}$ MF achieved the best accuracy for the **CDE - NO** dataset.

**Table 3:**
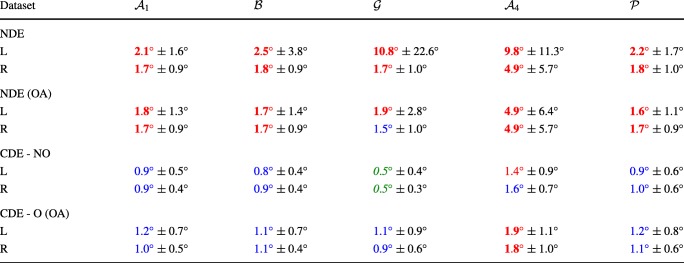
The average and standard deviation of accuracy for all datasets

The format is *mean* ± *standard deviation*. The accuracies are divided and color-coded into three categories; : 0°− 0.5°, : 0.51°− 1.5° and : $1.51^{\circ } - \inf ^{\circ }$. The (OA) indicates that the outlier correction algorithm has been used during the calibration

For the **NDE data** and **CDE - O** data (both with OA) where calibration data fixation inaccuracies are present, the accuracies for the $\mathcal {A}_{1}$, $\mathcal {B}$, $\mathcal {G}$ and $\mathcal {P}$ mapping functions are approximately the same while the $\mathcal {A}_{4}$ yields a considerably worse accuracy. The fact that the accuracies are worse for the **NDE** database than for the **CDE - O** database indicates that the true Nystagmus calibration errors are more severe than the simulated ones. If good accuracies are defined as being smaller than or equal to 0.5°, it is difficult to achieve good accuracy with inaccuracies in the calibration data.

### Calibration plane distortion

**Fig. 7:**
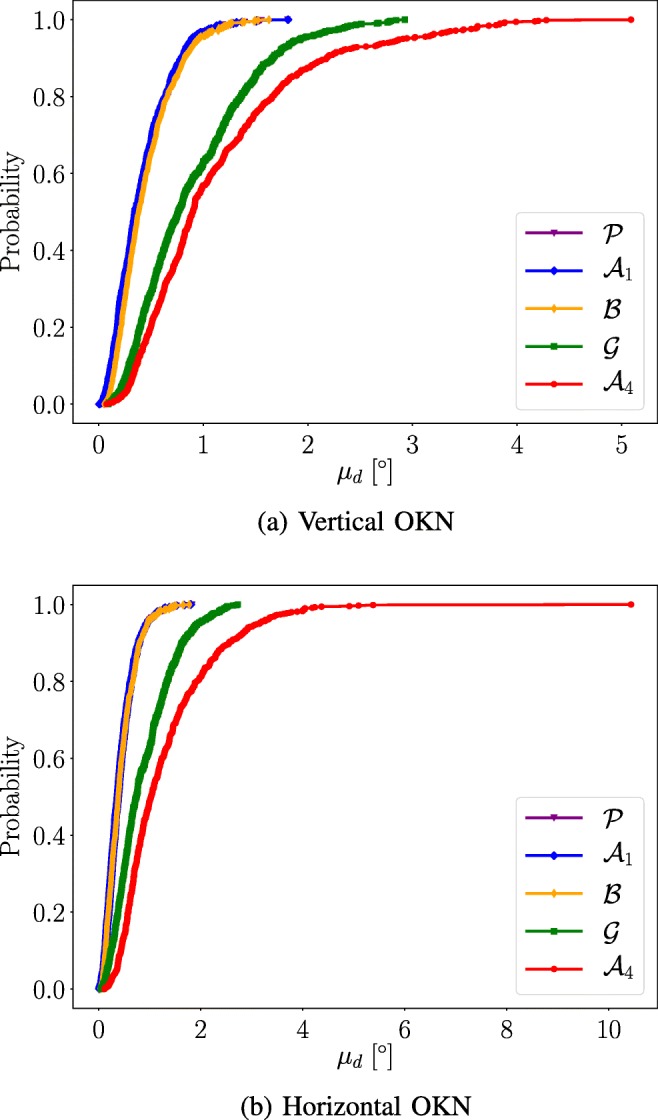
The calibration plane distortion plotted for the five mapping functions as cumulative distribution functions. The calibration plane distortion of the $\mathcal {A}_{1}$, $\mathcal {B}$ and the $\mathcal {P}$ are almost the same. The $\mathcal {G}$ and $\mathcal {A}_{4}$ polynomials performs worse compared to the three aforementioned MFs

The calibration plane distortion CDFs are presented in Fig. 7 and the *A*_*CDF*_ results are listed in Table 4. The differences between the results for the vertical and horizontal OKN data within each MF are small. The performance of the $\mathcal {A}_{1}$, $\mathcal {B}$ and $\mathcal {P}$ MFs are quite similar. The results for the other two MFs are worse. This is confirmed by Fig. 7.

**Table 4:** ***A***_***CDF***_ Results

Dataset calibration plane distortion (*μ*)	$\mathcal {A}_{1}$	$\mathcal {B}$	$\mathcal {G}$	$\mathcal {A}_{4}$	$\mathcal {P}$
Vertical	0.61	0.57	0.29	0.23	0.59
Horizontal	0.59	0.57	0.31	0.18	0.58
Waveform robustness (*ξ*)					
Vertical	0.91	0.82	0.64	0.36	1.00
Horizontal	0.93	0.85	0.80	0.43	1.00

The $\mathcal {A}_{1}$ MF generates the best calibration plane distortion scores whereas the $\mathcal {P}$ MF generates a perfect waveform robustness score (1.0). The difference in *μ*-value for the $\mathcal {A}_{1}$, $\mathcal {B}$ and $\mathcal {P}$ MF are quite small. The analysis was conducted on OKN data

### Waveform robustness

**Fig. 8:**
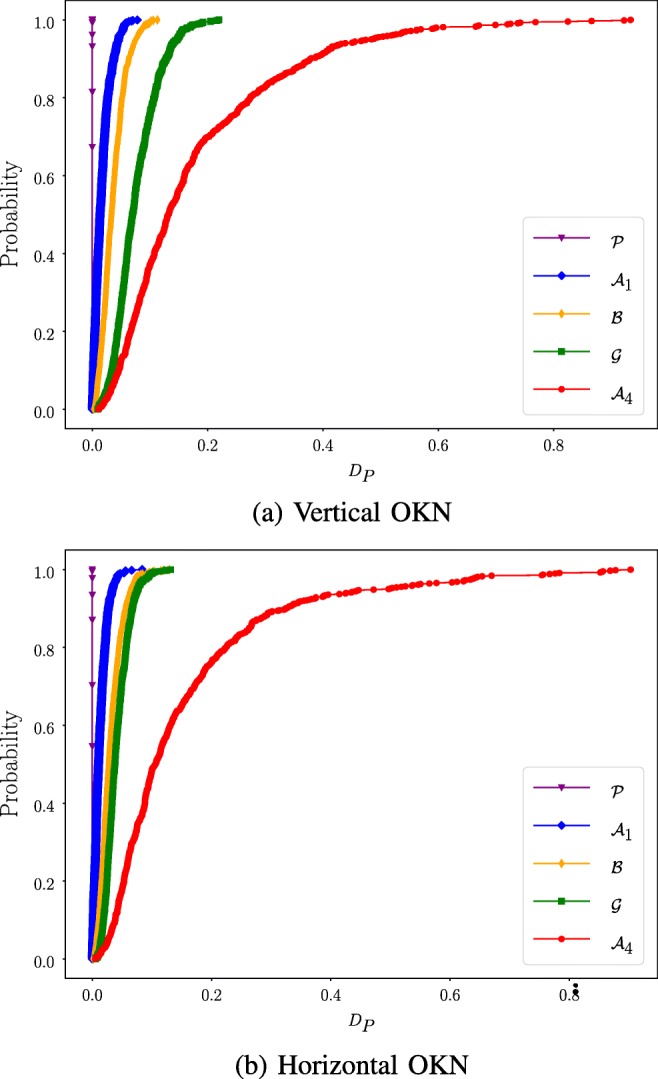
Waveform Robustness CDF. The vertical and horizontal OKN data CDF:s for waveform robustness. The $\mathcal {P}$ generates the best results and the $\mathcal {A}_{4}$ generates the worst results

The waveform robustness CDFs are presented in Fig. 8 and the corresponding *A*_*CDF*_ results are presented in Table 4. The results in Fig. 8 show that the Procrustes calibration method performs the best and the $\mathcal {A}_{4}$ performs the worst for both the vertical and the horizontal OKN tasks. This is quantified in Table 4. The waveform robustness seems to be linked to the non-linearity of the MF; a higher degree of non-linearity causes worse waveform robustness performance and vice versa.

### Waveform robustness and accuracy examples

**Fig. 9:**
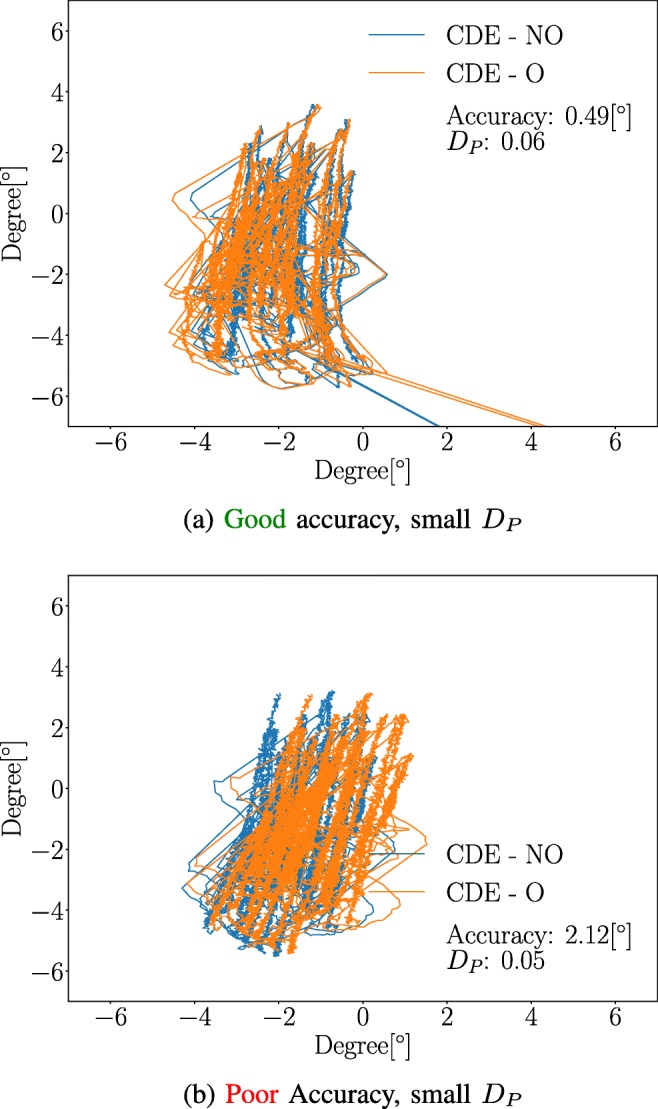
Small *D*_*P*_. Examples of waveforms where the **CDE - O** estimation is similar to the **CDE - NO** estimation. The probabilities of the *D*_*P*_-values 0.06 and 0.05 are *Prob*(*D*_*P*_ > 0.06) ≈ 0.62 and *Prob*(*D*_*P*_ > 0.05) ≈ 0.73 for **CDE - O** dataset, respectively. As illustrated by the plots, it is possible to achieve good waveform robustness even if the accuracy is . The $\mathcal {G}$ MF was used for all estimations. The analysis was conducted on OKN data

**Fig. 10:**
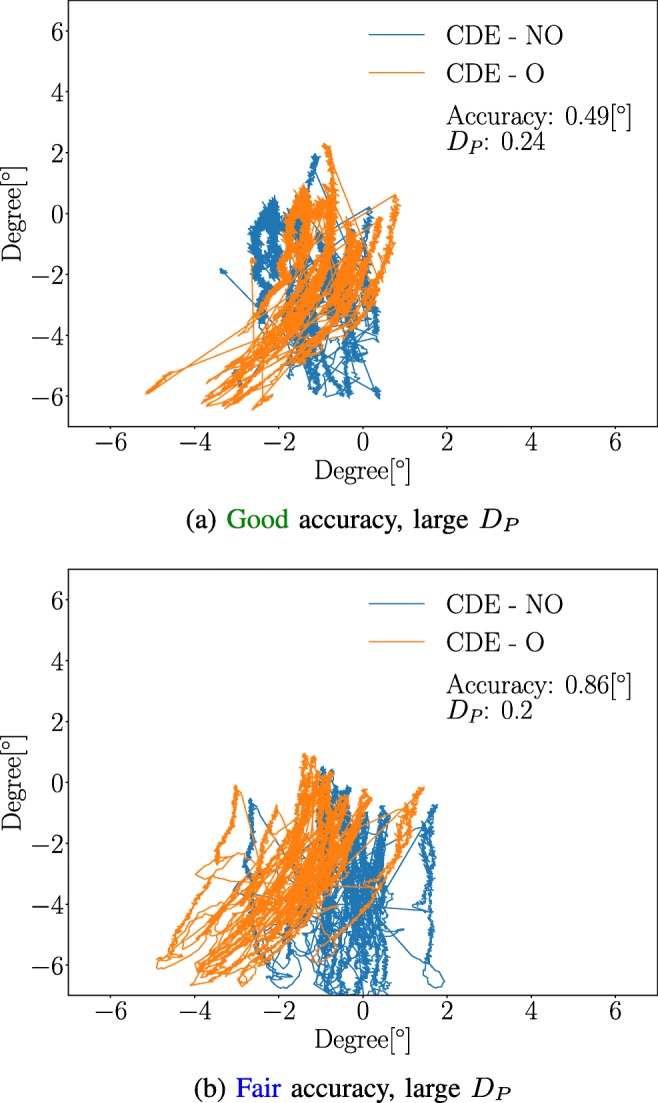
Large *D*_*P*_. Examples of data generating  accuracy 10a and 10b accuracy, but with poor waveform robustness values. The probabilities of the *D*_*P*_-values of 0.2 and 0.24 are *Prob*(*D*_*P*_ > 0.2) = 0.01 and *Prob*(*D*_*P*_ > 0.24) < 0.01 for **CDE - Offset** dataset, respectively. The $\mathcal {G}$ MF was used for all estimations. The analysis was conducted on OKN data

A few examples illustrating the relationship between accuracy and waveform robustness for the $\mathcal {G}$ MF are presented in Figs. 9 and 10. As can be seen in Fig. 9, it is possible for an MF to produce small waveform robustness values, *D*_*P*_ = 0.05, with a relatively large accuracy value, 2.12°. On the other hand, Fig. 10 illustrates that a  accuracy does not guarantee a small waveform robustness value. A *D*_*P*_ value larger than 0.2 is high, since only 1 % of the waveforms generates a higher value in the **CDE - O** dataset. All waveforms estimations were made using the $\mathcal {G}$ MF.

## Discussion

In this paper, we investigated the suitability of commonly used calibration mapping functions for data from people with nystagmus and proposed a new approach for calibration of these participants. The new method utilises an outlier correction algorithm based on the experiment geometry and calibrates the eye tracker using Procrustes analysis. Our method was compared to different calibration MFs previously used in nystagmus research. Accuracy and Procrustes distance were used to study the properties of the various MFs. Procrustes distance was used to study waveform robustness, i.e., how well waveform PoR data can be repeated within the same participants despite fixation inaccuracies during the calibration, and calibration plane distortion, i.e., how close, in absolute terms, data with simulated fixation inaccuracies were to data without simulated fixation inaccuracies. Data from people with nystagmus (**NDE**), visually healthy participants (**CDE - NO**) and participants with simulated fixation inaccuracies (CDE - Offset) were included in the study.

The accuracy data show that there is little difference between the $\mathcal {A}_{1}$, $\mathcal {B}$, $\mathcal {G}$ and $\mathcal {P}$ MFs for the **NDE** and **CDE - O** when using the outlier algorithm. However, when studying the calibration plane distortion presented in Fig. 7 and Table 4 it becomes apparent that the $\mathcal {G}$ polynomial performs worse compared to the $\mathcal {A}_{1}$, $\mathcal {B}$ and $\mathcal {P}$ MFs. This observation is likely explained by poor performance on interpolated data (the OKN dataset) by the $\mathcal {G}$ polynomial. The calibration plane distortion thought as an accuracy measure for interpolated data, using the **CDE - NO** as reference. Finally, the results from the waveform robustness in Fig. 8 show that the $\mathcal {P}$ MF has the best performance Since the Procrustes calibration method is based on linear operations only, the waveform robustness is 1.0 by default. The performances of the other MFs are ordered by their non-linearity; the more non-linear, the worse performance. The overall results show that it is not beneficial to use non-linear mapping functions when working with difficult to calibrate participants. Therefore, Procrustes analysis is the best choice when repeatable calibrations are desirable.

The outlier correction algorithm improved the validation accuracies in all cases. This suggests that there is a potential value in modelling the experiment geometry. Even though our results show that the accuracy alone is not a reliable measure for evaluation of an MF it is still desirable to improve the accuracy as long as it does not affect other properties, such as the waveform. It should be noted that if the distribution of the calibration targets is different from the one presented in this paper, the algorithm needs to be adapted for the specific target constellation. One could try to find the geometric relationship between data and targets for calibration target distributions as well, but that would likely demand a more in-depth analysis of the geometry of the experimental setup. The threshold for detecting an outlier, described in Stage I of the outlier correction algorithm in “The outlier correction algorithm”, is an important parameter for the correction performance. This parameter reflects the maximum deviation that is accepted from the theoretical horizontal distribution of the calibration data. As can be seen in Fig. 4, the foveation position varies spontaneously for people with nystagmus. If the threshold value is set too low, there is a risk to affect the structure of the calibration data. On the other hand, if the threshold is set too high, there is a risk to not detect outliers in the data.

The reason why accuracy is not considered as a good indicator of calibration performance for people with nystagmus are the following: 1) It is difficult to know if the validation data were recorded when the participant looked at the corresponding validation target. The accuracy analysis does not make sense if the participant did not look at the presented target, since the entire point of the validation is to test how well the mapping function transforms PCRV data to some known position. Since gaze estimation is dependent on the calibration, it is not possible to know if poor validation results originate from the calibration or the validation. 2) Data distortion effects, as shown in Fig. 10a, may occur even if the accuracy is considered to be . This is a problem because one will think that the calibration went well, when in reality gaze data do not correspond to the actual eye movements generated by the participant. However, accuracy is a good measure in the sense that it is a unit (degree) that can be compared between recordings and systems.

The distance measure was included to complement the accuracy and it was used to study how the waveform is affected by the calibration. A problem with the calibration plane distortion and waveform robustness measures is that the value may be difficult to interpret. In this paper, we have computed them on the same PCRV dataset for each mapping function, which makes it possible to compare the distance values between the MFs. The results can only be used to find *that* there are differences in the waveform, not the nature of these differences. For the nystagmus case, more specific differences such as foveation duration, amplitude, frequency and the nystagmus waveform, are of interest but are not possible to find using *D*_*P*_.

The **CDE - O** used in this work is likely not representative of fixation inaccuracies caused by nystagmus, which the results also indicate; the accuracy of **CDE - NO** is better compared to that of the **NDE**. The idea of making random errors of fixed magnitude does have its limitations and a continuous distribution may possibly be a more realistic representation of the fixation errors for some participants. Signal (1) in Fig. 4 shows that it is possible for the position after the fast phase to vary as much as 4° between cycles. The fixation errors introduced in the CDE - O database are therefore considered reasonable.

The creation of the **CDE - NO** and **CDE - O** databases serves a useful purpose in the sense that we have created two identical PCRV datasets, but with different estimates of the mapping functions. This allowed us to study differences between the tested calibration mapping functions. It is not possible to turn off the nystagmus oscillations for the affected patients, causing this analysis to be impossible to carry out for nystagmus data, since there is no reference waveform to compare the estimations with.

In this work, we tested the EyeLink 1000 Plus system, which is frequently cited in nystagmus research. The applicability of the proposed method for other eye trackers has not been studied.

Finally, the calibration data selection has not been central to the analysis in this paper. It is reasonable to assume that a poor calibration data selection method does have a negative impact on the PoR results, especially considering the results presented in this paper. The adjustments to Dunn’s method (Dunn, 2014) may have influenced the results in this paper. But based on the data we recorded, the method adjustments are considered reasonable. An updated version of the method has recently been developed (Dunn et al., 2018). This method may further improve the accuracy of the algorithm. It should also be noted that the calibration data selection implemented in this work is designed for nystagmus with foveation periods or at least waveforms with a distinct fast phase. As can be seen in Fig. 4, there were no pendular waveforms present in this dataset. For pendular nystagmus waveforms, it is, however, still possible to use the method since the outlier correction algorithm estimates missing data. It is, however, necessary to have at least three recorded data points, one in each row and one in each column, in order for the algorithm to work.

## Conclusion

The Procrustes analysis calibration method was shown to be the best when working with data from participants who have a decreased ability to fixate their gaze during the calibration. The principal difference between the Procrustes calibration method and the other investigated methods was the ability to generate repeatable waveform estimations regardless of the calibration recording condition. The choice of calibration mapping function may have a significant impact on the resulting eye movement estimations, which in turn may decrease the reliability of subsequent data analysis.
